# Acute toxicity of peroxy sulfonated oleic acids (PSOA) to freshwater aquatic species and sludge microflora as observed in laboratory environments

**DOI:** 10.1186/s12302-015-0054-5

**Published:** 2015-09-30

**Authors:** Stephan Solloch, Nathan Pechacek, Bridget Peterson, Magdalena Osorio, Jeffrey Caudill

**Affiliations:** 1 Ecolab, Monheim, Germany; 2 Ecolab, St. Paul, MN USA; 3 Ecolab, Naperville, IL USA

**Keywords:** Laboratory aquatic study, Acute aquatic toxicity, Peroxy sulfonated oleic acids (PSOA), PNEC, *Oncorhynchus mykiss*, *Daphnia magna*, *Pseudokirchneriella subcapitata*

## Abstract

**Background:**

Peroxy sulfonated oleic acids (PSOA) is a novel surfactant peracid. The commercial applications of PSOA result in the chemical primarily being disposed of via industrial waste water effluent. Given this manner of disposal, it is important to understand the aquatic hazards of the chemical to better assess the risk posed to aqueous environments. Acute aquatic toxicity laboratory experiments were performed to evaluate aquatic hazards and were conducted according to standard OECD test guidelines with rainbow trout (*Oncorhynchus mykiss*), water fleas (*Daphnia magna*) and algae (*Pseudokirchneriella subcapitata*). In addition, microbial toxicity was evaluated in activated sludge obtained from a domestic sewage treatment facility.

**Results:**

Lethal concentration in 50 % of test species (LC_50_) and effect concentration in 50 % of test species (EC_50_) values for PSOA ranged from 0.75 to 5.44 mg/L, representing a relatively small range spanning less than an order of magnitude. No observed effect concentration (NOEC) and lowest observed effect concentration (LOEC) ranges were also relatively small, with ranges of 0.25–1.66 and 0.5–3.6 mg/L, respectively. The EC_50_, LOEC and NOEC values for microbial toxicity were 216, 60 and 20 mg/L, respectively. Predicted no effect concentrations (PNEC) for aqueous media were based on the 96-h LC_50_ (0.75 mg/L) for *O. mykiss*, the organism displaying the greatest sensitivity to PSOA. These values were derived for freshwater, marine water and intermittent releases to water and ranged from 7.5 × 10^−5^ to 7.5 × 10^−3^ mg/L. A sewage treatment plant PNEC of 2 mg/L was derived based on an activated sludge 3-h NOEC of 20 mg/L.

**Conclusion:**

These values, along with the anticipated environmental fate and transport for PSOA, were considered in assessing the overall aquatic risk posed by this chemical. Despite the relatively high acute aquatic hazards for PSOA, environmental modeling suggests the overall risk of PSOA to aqueous environments is low based on its anticipated uses. This conclusion is consistent with the significant processing of industrial wastewater by onsite or municipal wastewater treatment facilities prior to release to the environment.

## Background

**Fig. 1:**
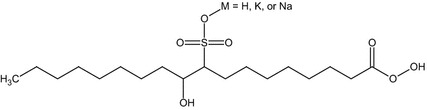
Chemical structure of PSOA

Peroxy sulfonated oleic acids (PSOA) is the common name of the substance “reaction product of sulfonated oleic acid potassium salt, hydrogen peroxide and sulfuric acid” (IUPAC name) (see Fig. 1). The official CAS entry is “9-Octadecanoic acid (9Z)-, sufonated, oxidized, potassium salts” (CAS number 1315321-94-8). As described in Pechacek et al. [13], PSOA is an organic peroxide that is characterized by the presence of one or more oxygen–oxygen bonds and is derived by reacting sulfonated oleic acid and hydrogen peroxide under acidic conditions [13, 14]. The oleic acid used in the production of PSOA is derived from renewable feedstocks such as plant oils and animal tallow [13]. Due to the variability inherent in the natural feedstock used to obtain oleic acid, PSOA subsequently falls into the regulatory definition of an unknown or variable composition, complex reaction products or biological materials (UVCB) substance [5, 13]. The resulting equilibrium mixture contains peroxy sulfonated acids, hydrogen peroxide, water and residual acid.

PSOA is a novel surfactant peracid developed to alleviate challenges with current peracid technology. It is commercially used as an antimicrobial, bleaching or coupling agent. Food and beverage industries primarily use PSOA as a coupling agent to clean and sanitize processing equipment [13]. PSOA is commonly available commercially as a concentrate due to its ability to maintain equilibrium longer as a concentrate than in diluted solutions. When PSOA comes into contact with organic matter such as milk or blood, it rapidly decomposes to its precursor reactants. Additionally, it rapidly decomposes when dried on a surface due to the water loss that shifts the equilibrium of the PSOA mixture [7]. PSOA is readily soluble in water with an aqueous solubility of ≥43.8 g/L at approximately 20 °C [15]. However, it is hydrolytically unstable and significant hydrolysis occurs at environmentally relevant pH values (4, 7 and 9) [2].

Disposal of PSOA primarily occurs in industrial wastewater effluent. Given this method of disposal, it is important to characterize the aquatic hazards of PSOA. This publication summarizes the results of four studies: a 96-h continuous flow study with rainbow trout (*Oncorhynchus mykiss*), a 48-h immobilization test with *Daphnia magna*, a 72-h algal growth inhibition test with *P. subcapitata* and a 3-h respiration inhibition test (ASRIT) with activated sludge. Given the known environmental fate and transport of PSOA in aqueous environments, the overall goal is to discuss the aquatic hazards identified in their appropriate risk context.

## Methods

### Test item

100 % UVCB substance PSOA was synthesized by Ecolab chemists and served as the test article. PSOA was stored at −20 °C and thawed prior to preparation of stock solutions, and was described as a viscous, pasty white or yellowish liquid. The PSOA used in all described tests originated from the same manufactured batch.

### Experimental organisms

To determine the aquatic toxicity of PSOA, acute toxicity experiments were performed on three different freshwater species: rainbow trout (*O. mykiss*), water flea (*D. magna*) and algae (*P. subcapitata*). Acute lethality was evaluated for the fish (96 h), acute immobilization for the *D. magna* (48 h) and effects to growth rate and yield for algae (72 h). In addition, the toxicity of PSOA to indigenous microflora found in activated sludge of a domestic sewage treatment facility was assessed.

#### Fish

Fish obtained from Selcoth Fish Farm in Moffat, Scotland were used for the initial and repeat range finding tests, while fish from Brow Well Fisheries Ltd., Skipton, England were used in the definitive test. Fish measured 4–6 cm in length and were free from any apparent malformation or poor health. All fish were acclimated to laboratory conditions for a minimum of 12 days prior to commencement of the study. During holding, fish were fed daily. Twenty-four hours prior to initiation of the study, food was withheld from the fish and no food was provided during the study.

#### *Daphnia magna*

*Daphnia* were obtained from the Laboratory of Hydrobiology (Central Agricultural Office, Directorate of Plant and Soil Protection), Hungary. All daphnids were female and under 24 h old. *Daphnia* were bred and acclimatized in the laboratory under similar temperatures, light conditions and water quality as were used in the definitive test. *Daphnia* were fed a centrifuged green alga suspension during holding and no food was provided during the study.

#### Algae

Algae were supplied by the SAG: Collection of Algal Cultures, Institute of Plant Physiology, University of Göttingen, Germany. The stock cultures were small algal cultures that were planted on agar regularly and transferred to fresh medium at least once every 2 months. The pre-culture was intended to give an amount of algae suitable for the inoculation of test cultures. The pre-culture was prepared with Algal Mineral Salts Culture Medium, incubated under the conditions of the definitive test and used when growing exponentially, which normally occurred after an incubation period of 3 days.

#### Activated sludge

Activated sludge was obtained from Totnes Sewage Works, Totnes, Devon, UK 1 day prior to the start of the definitive test. This sludge was used predominantly in the treatment of domestic sewage. The activated sludge was settled and the supernatant decanted. The settled sludge was incubated with 50 mL of OECD synthetic sewage feed per liter of sludge per day and aerated at room temperature until testing commenced. The OECD synthetic sewage feed was prepared as described in OECD Test Guideline 209 [12].

### Aquaria and water quality

#### Fish

Fish were kept in 12-L (initial and repeat range finding tests) and 17-L (definitive test) tanks of reconstituted freshwater (RFW) according to the formula recommended by OECD Guideline 203 [9]. High-grade salts were dissolved in 500 L of deionized, reverse osmosis-grade water and this solution was metered into a flow of deionized water to produce reconstituted water. Reconstituted water was stored at 12 °C and constantly aerated. For the initial range finding test, the pH of the holding and test medium was adjusted to 5.0–6.5. For the repeat range finding test and the definitive test, a reduced pH was not required and the holding and test medium were maintained at a pH range of 6.0–8.5. Test vessels were kept within a temperature-controlled laboratory with the aim of achieving a temperature in the range of 13–17 °C. A light cycle of 16 h of light and 8 h of darkness was in operation throughout the test. Artificial daylight fluorescent tubes provided illumination.

#### *Daphnia magna*

*Daphnia* were kept in beakers containing approximately 40 L test medium. Reconstituted water (ISO medium according to OECD 202) was prepared by adding 25 mL from each of four stock solutions to 1 L of water [10]. It had an approximate total hardness of 249 mg/L CaCO_3_, a dissolved oxygen concentration of 8.31–8.81 mg/L, a pH of 7.50–8.08 and a temperature range of 19.6–20.5 °C. The temperature of the climate chamber was in the range of 19.2–20.6 °C, with 16-h light and 8-h dark cycles. Artificial daylight fluorescent tubes provided illumination.

#### Algae

Algae were kept in 250-mL Erlenmeyer flasks with 100 mL test medium. Water temperature in the flasks was in the range of 22.4–23.0 °C while the climate chamber had a temperature range of 22.1–23.9 °C. The pH was measured at the beginning and end of the study for each test concentration and the control, and it ranged from 5.99 to 8.57. Algal flasks were continuously illuminated. The light intensity at the position occupied by the algal cultures in the flasks was approximately 7947 lux, which was maintained by fluorescent lamps with a spectral range of 400–700 nm. The light intensity between test flasks did not vary in excess of ±15 %.

#### Activated sludge

The activated sludge contained a total filterable solid concentration of 8307 mg/L as determined on the day of testing. The pH of the sludge was measured as 5.5 and adjusted to 7.3 by the addition of 2 M sodium hydroxide. One drop of antifoaming agent, antifoam B emulsion, was dispensed in each flask after initial foaming was observed during aeration. The antifoam B emulsion contained 10 % active silicone. Flasks containing activated sludge were aerated at a temperature of 20 ± 2 °C.

### Experimental designs

#### Fish

Preliminary range finding testing was conducted to determine the appropriate concentrations of the test item for the definitive testing. Weighed amounts of PSOA (5.01, 10.12, 20.15 and 50.3 mg) were individually added to 1 L of RFW and ultrasonicated for 30 s to ensure solubility and homogeneity to create four nominal concentrations: 5, 10, 20 and 50 mg/L. The pH values for each solution were determined. Based on these values, the initial range finding test was conducted at 20 mg/L, as this test concentration had a pH (5.78) similar to the pH to which the fish were acclimating. The initial range finding test was conducted over a 96-h period under semi-static conditions, at nominal concentrations of 2.5, 5, 10 and 20 mg/L with an untreated control. Three fish per tank were used, with one tank per concentration and one control. Due to overt effects observed within 18 min at all test concentrations, the study was terminated and all fish were humanely euthanized to avoid additional stress. A repeat range finding test was conducted at 0.002, 0.02, 0.2 and 2 mg/L with an untreated control. Once again, three fish per tank with one tank per concentration and control were used. Test solutions were prepared daily by dilution of a 20 mg PSOA/L stock solution with RFW. Fish were transferred to freshly prepared test solutions at 24-h intervals.

The definitive 96-h test with fish was conducted at nominal PSOA concentrations of 0.0625, 0.125, 0.25, 0.5 and 1 mg/L with a solvent and untreated control and continuous flow conditions. Seven fish were randomly added to each tank at the start of the exposure phase. For determination of the PSOA concentration, samples were taken from the testing concentrations and the controls at −24, 0, 24 and 96 h. For each test concentration, RFW was passed from a header tank into a pre-exposure glass mixing vessel containing a magnetic follower. The nominal flow rate of RFW to each mixing vessel was 35 mL/min which equated to five volume replacements per tank every 24 h. The water was mixed with a continuous flow of stock solution of PSOA in acetone or acetone alone for the solvent control. The stock solutions were contained in Becton–Dickinson syringes equipped with a 16-gauge Teflon syringe infusion tube and delivered using Medfusion Syringe Infusion Pumps, Model 2001. Stock solutions at the appropriate concentration were delivered to the mixing vessel at a rate of 0.21 mL/h where they were mixed with the dilution water. The test solutions flowed through tubing directly to the appropriate tank at a continuously controlled flow rate. Excess solution was siphoned off with an overflow tube. The delivery of solutions to the solvent and non-solvent control tanks was identical to the treated tanks but excluded PSOA. The solvent control received acetone only from the infusion pump, which was mixed with RFW and the non-solvent control received only RFW. Dose solution delivery rates were confirmed daily for each tank by collecting the outflow of dose solution over a timed interval and measuring the volume. Temperature, pH, conductivity, dissolved oxygen concentration and hardness of test water were also monitored throughout the study. During the in-life phase of the test, fish were observed at 1, 4, 24, 48, 72 and 96 h. Incidence of fish death was recorded and abnormalities noted. At the conclusion of the study, the length and weight of surviving fish were recorded.

#### *Daphnia magna*

For the range finding test for *Daphnia*, nominal PSOA concentrations of 0.01, 0.1, 1, 10 and 100 mg/L were prepared by appropriate dilution of a stock PSOA solution. The stock solution consisted of 100 mg PSOA/L and was prepared by dispersing the test item in ISO medium and then shaking for 30 min. Non-dissolved test material was separated by centrifugation. For the range finding test, ten daphnids for each test concentration and control were exposed for 48 h. Two replications of this test were conducted.

Based on the observations for the range finding test, five test concentrations in a geometric series with a separation factor of two and one control was used in the 48-h definitive test. The nominal concentrations were 0, 2.5, 5.0, 10.0, 20.0 and 40.0 mg/L. Preparation of the stock PSOA solution and test solutions occurred in the same manner as the range finding test. For determination of the PSOA concentration, samples were taken from the testing concentrations and the control at the start and end of each water renewal period. A semi-static water renewal method was chosen with a renewal frequency period of 24 h. Twenty *Daphnia* were exposed to each test concentration, with the twenty animals divided into four groups of five. Each group resided in approximately 40 mL of test medium. *Daphnia* were observed at 24- and 48-h intervals and were considered immobile when they were unable to swim after 15 s.

#### Algae

A range finding test was conducted in which algae were exposed for 72 h to nominal concentrations of 0.01, 0.1, 1, 10 and 100 mg/L. A PSOA stock solution and test solutions were prepared for the range finding and definitive tests as described in “Daphnia magna” section. Based on cell number counts from the range finding study, six test concentrations in a geometric series with a separation factor of two and one control was used in the 72-h definitive test. The nominal concentrations were 0, 0.4, 1.0, 2.6, 6.4, 16.0 and 40.0 mg/L. Introduction of algae into the 250-mL Erlenmeyer flasks occurred at 0 h by inoculation of 0.1 mL algal biomass (10^7^ algal cells/mL) into 100 mL test solutions. The algal cells were taken from an exponentially growing pre-culture established 4 days prior to the start of the definitive study. The initial cell density was about 10^4^ cells/mL in each test flask. There were three replicates per test concentration and six replicates in the untreated control. During incubation the flasks were stored on an orbital shaker and continuously shaken. Algal cell numbers and morphology were assessed at 24, 48 and 72 h. Cell number was determined by manual cell counting using a microscope with a counting chamber. Morphology was assessed microscopically.

#### Activated sludge

The definitive study ran in two sets of 3-h exposure periods in the course of 1 day. The first set consisted of three PSOA concentrations, five reference flasks and four control flasks. The second set consisted of two PSOA concentrations, five reference flasks and three control flasks. Nominal concentrations of 6, 20, 60, 200 and 600 mg PSOA/L were prepared in replicates of five, in addition to a control. Flasks of the reference substance, 3,5-dichlorophenol (3,5-DCP) were prepared at nominal concentrations of 3.2, 10, 32 and 100 mg/L. 3,5-DCP was used as a reference substance given its known inhibitory effect on respiration, as well to ensure that the batch of sludge used in the test showed a representative level of sensitivity. An abiotic flask at 100 mg/L and a control were also prepared.

Each flask contained 3.2 mL of synthetic sewage and 18 mL of activated sludge to give a final solids concentration of 1500 mg/L (with the exception of the abiotic flask), an appropriate quantity of either PSOA or 3,5-DCP stock solution and reverse osmosis water to give a final flask contents volume of 100 mL. After foaming was observed in the first aerating flasks, one drop of antifoaming agent was added to each test flask. The pH of each flask was measured at the beginning and end of the test. The pH of the PSOA stock solution was adjusted to 6.6 before use and the reference substance stock solution had a pH of 7.3.

Flasks were established in batches of six and aerated at 20 ± 2 °C for 3 h. Each batch included a control flask and five test or reference substance flasks. The temperatures of the flask contents were measured at the end of the 3 h aeration using a mercury-in-glass thermometer. The respiration rate of each flask was measured after 3 h and compared with the mean respiration rates of the control flasks. The rate of oxygen uptake was measured in glass sample tubes into which microcathode oxygen electrodes were inserted. The electrodes were connected to an interface unit, which converted the current produced by the electrodes into dissolved oxygen readings. These readings were transferred to a computer that calculated the respiration rate in each flask over the linear part of the curve and compared it to the mean of the control cultures. The rates of oxygen uptake were expressed as mg/L/h.

### Analytical measurements

#### Fish

An analytical procedure for the determination of PSOA in Tap Water formulations has been developed and validated by Charles River [3]. Formulation prepared at 2.5 µg/mL was found to be not stable when stored at ambient laboratory temperature in the dark. Formulation prepared at 150 µg/mL was found to be stable for 12 h when stored at ambient laboratory temperature in the dark. Due to the instability of the PSOA formulations in Tap Water the stability of PSOA in acetone was investigated. The formulation and analytical procedures were found to be satisfactory for a formulation prepared at 1.00 mg/mL in acetone which could be used for a flow through test in the ecotoxicology study.

In the range finding tests, analytical samples were collected in duplicate from the test concentrations at −24, 0, 24 and 96 h to assess stability. For the definitive test, duplicate samples were collected at the start of the study and after 72 h. Samples were collected from expired test media after 24 and 96 h. Sample analysis generally occurred on the same day as sampling. In cases where this was not feasible, the samples were stored under suitable conditions until analysis could take place.

#### *Daphnia magna*

Analytical samples were collected from the test concentrations and the control at the start and at the end of each water renewal period. Samples were analyzed directly using a photometric method in a manner similar to the fish study.

#### Algae

Analytical samples were collected from the test concentrations and the control at the start of the test and 24-h intervals thereafter. For these daily measurements, one extra replicate was used in each test concentration that was treated the same as the replicates used for determination of the algal cells. In a manner similar to the fish and *Daphnia* studies, samples were analyzed directly after sampling using a validated photometric method.

#### Activated sludge

No analytical measurements were taken during the study. Only nominal concentrations were used.

### Computational and statistical analysis

#### Fish

Median lethal concentrations (LC_50_) values were estimated by taking the arithmetic mean of the 0 and 100 % mortality concentrations. No observed effect concentration (NOEC) values were based on both mortality and observed effects.

#### *Daphnia magna*

NOEC, lowest observed effect concentration (LOEC) and effective concentrations for the 50th and 100th percentile values (EC_50_ and EC_100_) were determined for the definitive test. NOEC, LOEC and EC_100_ were identified directly from the data, while EC_50_ values were calculated by probit analysis with 95 % confidence limits.

#### Algae

The inhibition of algal growth was determined from the average specific growth rate and yield using the following equations:

$$ {\text{Average}}\,\,{\text{specific}}\,\,{\text{growth rate}}\,\,(\mu ):\mu_{i - j} = \frac{{\ln X_{j} - \ln X_{i} }}{{t_{j} - t_{i} }} + ({\text{day}}^{ - 1} ) $$

where *µ*_*i*−*j*_ is average specific growth rate from time *i* to *j*; *X*_*j*_ is biomass at time *j*; *X*_*i*_ is biomass at time *i*.

$$ {\text{Percent}}\,\,{\text{inhibition}}\,\,{\text{of}}\,\,{\text{growth}}\,\,{\text{rate}}\,\,(\% I_{r} ):\% \,\,I_{r} = \frac{{\mu_{C} - \mu_{T} }}{{\mu_{c} }} \times 100 $$

where *µ*_*C*_ is mean value for average specific growth rate in the control group; *µ*_*T*_ is average specific growth rate for the treatment replicate.

$$ {\text{Percent}}\,\,{\text{inhibition}}\,\,{\text{in}}\,\,{\text{yield}}\,\,(\% I_{y} ):\% \,\,I_{y} = \frac{{Y_{C} - Y_{T} }}{{Y_{C} }} \times 100 $$

where *Y*_*C*_ is mean value for yield in the control group; *Y*_*T*_ is value for yield for the treatment group.

Mean values and standard deviations of cell concentrations were calculated for each treatment at 0, 24, 48 and 72 h. The percent inhibition of growth rate (*µ*) and yield (*y*) was also calculated using Excel for Windows software. The EC_50_ values for growth rate and yield and their associated confidence limits were calculated using probit analysis based on measured geometric mean concentrations. For the determination of NOEC and LOEC, the calculated mean growth rate and yield at the test concentrations were tested on significant differences to the control values by Bonferroni *t* test. TOXSTAT software was used to evaluate the normal distribution of the rate and its homoscedasticity. Using EC_50_, NOEC and LOEC values were identified for algal growth rate and yield.

#### Activated sludge

The respiration of the flasks dosed with the PSOA or 3,5-DCP were expressed as percentages of the respiration rate of the control flasks and were derived as follows:

$$ {\text{Percent}}\,\,{\text{inhibition}}:\% \,\,I = \left[ {1 - \left[ {\frac{{{\text{Respiration }}\,\,{\text{rate}}\,\, {\text{of}}\,\,{\text{test}}\,\,{\text{flask}}}}{{{\text{Mean}}\,\, {\text{respiration}}\,\, {\text{rate }}\,\,{\text{of}}\,\, {\text{control }}\,\,{\text{flasks}}}}} \right]} \right] \times 100 $$

The effective concentrations (EC) for the 20th, 50th and 80th percentiles, as well as the NOEC for respiration inhibition, along with their 95th confidence intervals, were calculated by linear interpolation using the US EPA program ICPIN (Version 2, June 1993).

## Results

### Range finding tests

#### Fish

In the repeat range finding test, all fish in the highest dose group (2 mg/L) experienced difficulties in respiration within 1 h. Therefore, all fish were removed and humanely euthanized to avoid additional stress. Fish at all other test concentrations (0.002, 0.02 and 0.2 mg/L) appeared active and healthy throughout the exposure period with no abnormal effects or behavior. Measurement of all test solution quality parameters verified that pH, temperature, conductivity and dissolved oxygen concentration remained within acceptable limits throughout the duration of the test: pH range of 6.52–6.94, temperature range of 14–16.8 °C, conductivity range of 180.3–208 μS/cm and dissolved oxygen concentration range of 95–100 % air saturation value.

#### *Daphnia magna*

In the preliminary range finding test the *Daphnia* were considered immobile if they were unable to swim after 15 s. There were no immobile *Daphnia* observed at 48 h in the 0.01, 0.1 and 1 mg/L test groups. Six out of ten *Daphnia* were considered immobile in the 10 mg/L test group and all ten *Daphnia* were considered immobile in the 100 mg/L test group.

#### Algae

In the preliminary range finding test for algae, the nominal concentrations used were untreated, 0.01, 0.1, 1, 10 and 100 mg/L. The average of cell numbers at 72 h (×10^4^ cells/mL) was 67, 65, 61.5, 59, 43 and 1.2, respectively.

### Definitive tests

#### Fish

The definitive test results for fish are based on nominal concentrations of PSOA given that the test sample analysis confirmed PSOA levels were maintained at 99–100 % of nominal concentrations over the study duration. One dead fish was removed after 21 h of exposure to the highest dose (1 mg/L). The remaining fish in the same tank appeared lethargic. Two more dead fish were removed after 22.75 and 24 h, respectively. By 50.75 h, the remaining four fish were dead. After 72 h at the second highest dose (0.5 mg/L), all fish appeared to be respiring at an increased rate. All fish in both controls and at 0.0625, 0.125 and 0.25 mg/L appeared active and healthy throughout the test period. Fish surviving to 96 h were within the range 4.02–5.23 cm in length and 0.6984–1.7030 g in dry weight. No concentration-dependent growth effects were observed. Measurement of all test solution quality parameters verified that pH, temperature, conductivity and dissolved oxygen concentration remained within acceptable limits throughout the duration of the test: pH range of 6.41–7.15, temperature range of 16.0–16.9 °C, conductivity range of 194.8–208 μS/cm and dissolved oxygen concentration range of 74–97 % air saturation value.

#### *Daphnia magna*

The nominal concentrations used to test *Daphnia* were 0, 2.5, 5.0, 10.0, 20.0 and 40.0 mg/L with corresponding measured geometric mean concentrations of 0, 1.28, 2.69, 6.89, 15.61 and 32.91 mg/L, respectively. One hundred percent (100 %) of the daphnids were immobilized at 24 and 48 h at the two highest test concentrations (i.e., 15.61 and 32.91 mg/L). At 6.89 mg/L, 90 % of the daphnids were immobilized at 24 h and 100 % were immobilized at 48 h. At 2.69 mg/L, no immobilization was observed at 24 h; however, 33 % of the daphnids displayed immobilization at 48 h. No immobilization was observed at 1.28 mg/L or the control for the 24- and 48-h observations.

#### Algae

The nominal concentrations used to test algae were 0.4, 1.0, 2.6, 6.4, 16.0 and 40 mg/L. Geometric mean exposure concentrations were calculated for all nominal test concentrations except for the lowest two (i.e., 0.4 and 1.0 mg/L). The lowest two test concentrations were not within the measurable range of the photometric analytical method for the entire duration of the study and were not included in estimating EC_50_ values. For the third lowest test concentration, 2.6 mg/L, one measured concentration was below the limit of quantification (LOQ) of 0.25 mg/L and was assigned a value of one-half the LOQ (0.5 × LOQ) in calculating the geometric mean concentration. For the nominal concentrations of 2.6, 6.4, 16.0 and 40 mg/L, geometric mean exposure concentrations of 0.56, 1.66, 3.60 and 8.25 mg/L were calculated. In terms of the control, the algal cell density increased from a nominal level of 1 × 10^4^ cells/mL at 0 h to a mean value of 9.433 × 10^5^ cells/mL at 72 h, representing sufficient algal growth to pass the validity criteria of the assay. Microscopic evaluation of the treated algal cells showed thinner and smaller cells at the two highest test concentrations (3.60 and 8.25 mg/L) relative to the control. No algal effects were observed at the two lowest test concentrations.

#### Activated sludge

The validity criteria of a respiration rate was met by all control test flasks (at least 20 mg oxygen per one gram of activated sludge in an hour), with measured values ranging from 33.0 to 39.8 mg O_2_/g/h. The reference substance 3,5-DCP caused substantial inhibition of the respiration rate with a mean 3-h EC50 value estimated to be 2.6 mg/L. The percent inhibition for 3,5-DCP at 3.2, 10, 32 and 100 mg/L were 53.9, 76.1, 90.2 and 93.3, respectively. This was within the expected range of 2–25 mg/L, which indicated the sludge was responding accordingly and confirmed the viability of the sludge microflora [12]. The respiration rates in all the control flasks were within 15 % of each other. Therefore, the mean respiration rate of the control flasks associated with the reference substance and the relevant test concentration flasks was used to calculate the percent inhibition. The respiration rate of the abiotic control was negligible throughout the study.

### Identification of point-of-departure values

#### Fish

**Fig. 2:**
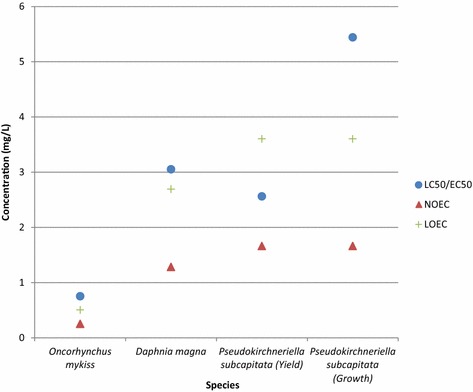
PSOA point-of-departure concentrations for *O. mykiss*, *D. magna* and *P. subcapitata*

Point-of-departure (POD) levels (i.e., LC_50_, LOEC, NOEC) for the definitive fish study are noted in Fig. 2. The study concentrations noted for the fish are the nominal concentrations. As noted in “Fish” section, mortality was observed at the highest test concentration (i.e., 1 mg/L). At the next lower concentration, 0.5 mg/L, an increased respiration rate was observed but no mortality. For the next lower concentration, 0.25 mg/L, no effects were observed. Based on these observations, the fish 96-h LOEC and NOEC are 0.5 and 0.25 mg/L, respectively. Due to the lack of fractional mortality, the 96-h LC_50_ was estimated using the arithmetic mean of the 0 and 100 % mortality concentrations resulting in a value of 0.75 mg/L with 95 % confidence intervals (CI) of 0.5–1 mg/L.

#### *Daphnia magna*

Effect levels for the definitive *Daphnia* study are noted in Fig. 2. The study concentrations noted for the *Daphnia* effective levels are the analytically confirmed test concentrations rather than nominal concentrations. The *Daphnia* 48-h NOEC was 1.28 mg/L as all organisms appeared to be swimming normally at this concentration and no immobilization effects were observed. The lowest 48-h test concentration resulting in immobilization effects was 2.69 mg/L (i.e., LOEC of 2.69 mg/L). The concentration resulting in 100 % immobilization was 6.89 mg/L (i.e., 48-h EC_100_ of 6.89 mg/L). The 48-h EC_50_ was calculated to be 3.05 mg/L (95 % CI 2.57–3.92 mg/L) (Fig. 2).

#### Algae

Effects levels for the definitive algal study are noted in Fig. 2. No effects were observed over 72 h at 0.56 and 1.66 mg/L (i.e., 72-h NOEC of 1.66 mg/L). Significant growth inhibition was observed over 72 h at ≥3.60 mg/L (i.e., 72-h LOEC of 3.60 mg/L). The 72-h EC_r_50 (i.e., EC_50_ for growth) was estimated to be 5.46 mg/L (95 % CI 4.98–5.99 mg/L) and 5.42 mg/L (95 % CI 4.77–6.16 mg/L) using the third and fourth highest test concentrations, respectively. The geometric mean of the EC_r50_ values is 5.44 mg/L. The 72-h EC_y50_ (i.e., EC_50_ for yield) was estimated to be 2.79 mg/L (95 % CI 2.62–2.97 mg/L) and 2.35 (95 % CI 2.08–2.66 mg/L) using the third and fourth highest concentrations, respectively. The geometric mean of the EC_y50_ of values is 2.56 mg/L.

#### Activated sludge

The NOEC and LOEC were determined to be 20 and 60 mg/L, respectively. The 3-h EC_20_, EC_50_ and EC_80_ values were calculated to be 46 mg/L (95 % CI 37–73 mg/L), 216 mg/L (95 % CI 187–252 mg/L) and 504 mg/L (95 % CI 480–525 mg/L), respectively. All POD values are based on nominal concentrations.

## Discussion

PSOA was developed as a surfactant peracid to alleviate challenges with current peracid technology. Given its commercial applications, the primary disposal mode for PSOA is via industry wastewater effluent, which raises the importance of understanding whether the substances pose a risk to the aquatic environment. Evaluation of acute aquatic toxicity endpoints is a critical first step in evaluating the aquatic hazards of PSOA for risk assessment given its primary mode of disposal.

The aquatic toxicity studies described in this article were performed according to standard OECD test guidelines, where applicable. Given the reactivity of PSOA, significant effort was expended to confirm nominal test concentrations with analytical measurements, with the exception of the activated sludge assay. In reviewing the acute toxicity results, LC_50_ and EC_50_ values for fish, *D. magna* and algae ranged from 0.75 to 5.44 mg/L, representing a relatively small range of less than an order of magnitude. However, the EC_50_ value calculated for sludge was appreciably higher at 216 mg/L. It is unclear why the aquatic species were more sensitive to PSOA than the sludge microflora; however, the rapid reactivity and subsequent attenuation of PSOA once it comes into contact with organic matter may help explain this observation.

In terms of acute NOECs (0.25–1.66 mg/L) and LOECs (0.5–3.6 mg/L) for the aquatic organisms, a similar observation of the LC_50_/EC_50_ ratio occurred; the NOEC and LOEC ranges were less than an order of magnitude apart. In terms of activated sludge, the NOEC (20 mg/L) and LOEC (60 mg/L) values were higher than the corresponding values for the test aquatic species. The small ranges for the POD values for the aquatic species indicates that PSOA has a relatively steep dose–response curve and rapid transitions from non-toxic concentrations to toxic levels over a small concentration range.

The mode-of-action for PSOA is not known, but of its four primary structural elements (i.e., hydroperoxide structure, acid component, sulfonate moiety and monohydroxy structure), the hydroperoxide structure is generally considered to be the defining component of the toxicity of PSOA [13]. In assessing the acute aquatic and microbial toxicity hazards of PSOA, insight can be garnered from evaluating acute toxicity studies conducted with similar hydroperoxides. Peracetic acid (PAA, CASRN 79-21-0) and *tert*-butyl hydroperoxide (TBHP, CASRN 75-91-2) are two representative hydroperoxides that can be used for comparison purposes (results shown in Table 1). From the table, it appears that PSOA has similar potency to PAA in terms of acute toxicity to *O. mykiss* but is slightly less potent to *D. magna* and *P. subcapitata* than PAA and appreciably less toxic to microorganisms. In comparison to TBHP, PSOA displayed greater toxicity to *O. mykiss* and *D. magna* but lower toxicity to *P. subcapitata* and sludge microorganisms. These comparisons are limited given the paucity of studies conducted for PSOA and TBHP, particularly relative to PAA. It is noted that much of the available aquatic information for peracids are based on industry-sponsored studies summarized in publicly available databases. There is relatively little published information for peracid in the peer-reviewed scientific literature. Therefore, a discussion on species sensitivity would be based on speculation regarding toxicity such as metabolic capacity of certain aquatic species that allow the bioactivation of PSOA resulting in, e.g., oxidative stress. Further the discussion above about the mode-of-action shows that structural similar chemicals show different species sensitivity regarding toxicity.

**Table 1:** Comparison of acute aquatic toxicity and microbial toxicity point-of-departure values for PSOA, PAA and TBHP

Species	Peracid chemistry					
	PSOA		PAA^a^		TBHP^b^	
	NOEC (mg/L)	LC_50_/EC_50_ (mg/L)	NOEC (mg/L)	LC_50_/EC_50_ (mg/L)	NOEC (mg/L)	LC_50_/EC_50_ (mg/L)
*Oncorhynchus mykiss* (96-h)	0.25 (*N* = 1)	0.75 (*N* = 1)	0.16–1.5 (*N* = 4)	0.91–2 (*N* = 4)	29.8 (*N* = 1)	29.61–56.88 (*N* = 2)
*Daphnia magna* (48-h)	1.28 (*N* = 1)	3.05 (*N* = 1)	0.035–<1 (*N* = 7)	0.035–1.1 (*N* = 7)	7 (*N* = 1)	14.1 (*N* = 1)
*Pseudokirchneriella subcapitata* (72-h growth rate)	1.66 (*N* = 1)	5.44 (*N* = 1)	0.084 (*N* = 1)	0.035–0.86 (*N* = 2)	0.22 (*N* = 1)	1.5 (*N* = 1)
*Pseudokirchneriella subcapitata* (72-h yield)	1.66 (*N* = 1)	2.56 (*N* = 1)	<1 (*N* = 1)	<1 (*N* = 1)	0.22 (*N* = 1)	0.8 (*N* = 1)
Activated sludge (3-h)	20 (*N* = 1)	216 (*N* = 1)	16.7 (*N* = 1)	5.1–38.6 (*N* = 2)	Not reported	17 (*N* = 1)^c^

^a^Referenced in OECD [11]

^b^Referenced in ECHA [6]

^c^30-min value rather than 3 h

The data for *O. mykiss* were used to derive an aquatic health benchmark for PSOA for use in a risk assessment given that this species displayed the greatest sensitivity to PSOA. The 96-h LC_50_ of 0.75 mg/L for *O. mykiss* was used to derive an aquatic predicted no effect concentration (PNEC) for PSOA. The activated sludge 3-h NOEC of 20 mg/L was used to derive a health benchmark for sewage treatment plants. Table 2 provides a description of the aquatic and sewage treatment PNEC values for PSOA.

**Table 2:** Aquatic and sewage treatment plant PNEC for PSOA

Environmental compartment	PNEC (mg/L)	Remarks and justification
Freshwater—PNEC aqua (freshwater)	0.00075^a^	Assessment factor: 1000 PNEC aqua (freshwater) equals lowest short-term LC50 value of 0.75 mg/L (fish) divided by assessment factor of 1000
Marine water—PNEC aqua (marine water)	0.000075^a^	Assessment factor: 10,000 PNEC aqua (marine water) equals lowest short-term LC50 value of 0.75 mg/L (fish) divided by assessment factor of 10,000
Intermittent releases to water—PNEC aqua (intermittent releases)	0.0075^a^	Assessment factor: 100 PNEC aqua (intermittent releases) equals lowest short-term LC50 value of 0.75 mg/L (fish) divided by assessment factor of 100
Sewage treatment plant—PNEC (STP)	2	Assessment factor: 10 PNEC STP equals 3 h NOEC of 20 mg/L divided by assessment factor of 10

^a^PSOA is not anticipated to be hydrolytically stable at these environmental concentrations

As noted earlier, the disposal of PSOA via industry wastewater effluent requires identification of aquatic hazards of this chemical. The acute aquatic and microbial toxicity testing of PSOA enables identification of these hazards, as well as derivation of various environmental health benchmarks (i.e., PNECs). However, another critical facet to assessing the overall risk of PSOA to aquatic environments is to consider its environmental fate and transport.

The stability of PSOA in aqueous environments under environmentally relevant pH values has been assessed following OECD Test Guideline 111 [2]. At pH levels of 4, 7 and 9, PSOA had hydrolyzed >10 % after 5 days with an estimated half-life of <1 year [2]. Based on these results, PSOA is considered hydrolytically unstable at environmentally relevant pH values. Experience with PSOA indicates that its stability in water is influenced by its aqueous concentration, with low ppm levels of PSOA showing less stability than higher concentrations (data not shown). This is important considering that in commercial applications of PSOA, the chemical is diluted prior to application and wastewater effluent containing PSOA is likely to be mixed with other facility wastewater lacking PSOA, further diluting the overall PSOA concentration prior to discharge and potentially accelerating chemical hydrolysis. Therefore, PSOA is not expected to persist in the environment.

Another important consideration is the microbial degradation potential of PSOA in aqueous environments. In an assessment following OECD Test Guideline 301B, PSOA biodegraded 56 % after 28 days with 40 % biodegradation observed by the Day 10 observation point (LAUS [8]. Though PSOA did not meet the OECD criteria for consideration as a “readily biodegradable” substance under the conditions tested, it did demonstrate appreciable aqueous biodegradability.

To gauge the hydrophilic nature of PSOA, EPI Suite software [KOWWIN Program (v1.68)] was used to estimate the Log Kow values for two of the primary components of PSOA, 10-hydroxy-9-sulfooctadecaneperoxoic acid and 10-hydroxy-9-sulfooctadecanoic acid [16]. Based on this work, a representative Log Kow of 3.12 was identified. The adsorption coefficient (Log Koc) can be used to estimate PSOA’s ability to bind to suspended sediment in water. Testing conducted in accordance with OECD Test Guidelines 121 identified Koc values ranging from 0 to 190,546 (Log Koc of un-retained to 5.28) [1]. To identify a representative value in this range, a Koc weighted average based on the percent peak area of the test solutions was calculated to be 1.06, resulting in a Log Koc estimate of 0.024. This representative value indicates a relatively low sediment binding potential overall for the PSOA mixture; however, select minor fractions of the PSOA mixture may readily bind sediment.

Based on the laboratory tests conducted for PSOA, it is suggested that the environmental aqueous fate of this substance would be to exist largely unbound in the water column with hydrolytic and biological factors degrading the chemical and limiting its availability to aquatic organisms. The rate of degradation may vary appreciably depending on the initial concentration of PSOA in the water column, and for some fractions of this mixture, persistence in the aqueous environment is possible. Additionally, a minor fraction of the PSOA mixture does displays appreciable sediment affinity which is anticipated to attenuate its toxicity and could increase its persistence in the aqueous environment.

As part of the requirements under the Registration, Evaluation, Authorization and Restriction of Chemicals (REACH), an environmental risk assessment was conducted using EUSES software to determine if the proposed uses of PSOA posed an unacceptable risk to aqueous environments. All risk characterization ratios (RCRs), which are ratios of the PNEC to the estimated environmental concentration (i.e., estimated environmental concentration/PNEC), were <0.01, indicating no unacceptable risk to the environment [4]. Therefore, despite notable acute aquatic hazards for PSOA when considering the acute toxicity values, no unacceptable risk to the aqueous environment is anticipated based on its use and other factors. This aligns with the qualitative assessment that PSOA in industry wastewater effluent would be further treated and diluted via a municipal waste water treatment facility prior to release to aquatic environments. Such treatment and dilution is anticipated to render PSOA below concentrations that pose a risk to native fauna or flora.

## Conclusion

PSOA is a novel surfactant peracid whose commercial use will result in disposal via industrial wastewater effluent. Given this manner of disposal, it is important to understand the aquatic hazards of the chemical to better assess the risk posed to aqueous environments. Laboratory testing of PSOA for representative freshwater fish, invertebrate and algal species displays a degree of toxicity that appears to align with other peracid chemistries, such as peracetic acid. However, PSOA displayed appreciably lower microbial toxicity in activated sludge relative to other peracids. Despite the relatively high acute aquatic hazards for PSOA, environmental modeling suggests the overall risk of PSOA to aqueous environments is low based on its anticipated uses. Such a conclusion aligns with the significant processing of industrial wastewater by municipal wastewater treatment facilities prior to release to the environment.
